# Understanding the Efficiency of Aluminum Coagulants Used in Dissolved Air Flotation (DAF)

**DOI:** 10.3389/fchem.2020.00027

**Published:** 2020-02-07

**Authors:** Ruben Miranda, Isabel Latour, Angeles Blanco

**Affiliations:** Department of Chemical and Materials Engineering, Faculty of Chemistry, Complutense University of Madrid, Madrid, Spain

**Keywords:** flocculation mechanisms, aluminum coagulants, dissolved air flotation (DAF), hybrid coagulants, focused beam reflectance measurement (FBRM), water reuse

## Abstract

This paper reports on the efficiency of five aluminum coagulants for the treatment of a paper mill wastewater by dissolved air flotation (DAF). The coagulants studied were: alum, a polyaluminum chloride coagulant of high aluminum content and intermediate basicity (PAC-MB), another with intermediate aluminum content and high basicity (PAC-HB), a polyaluminum nitrate sulfate of intermediate aluminum content and basicity (PANS) and one hybrid coagulant formed by the combination of PANS and a mixture of polyamines (PANS-PA). The influence of Al speciation on contaminants removal and the main flocculation mechanisms involved have been analyzed. High removal of suspended solids together with significant removal of dissolved and colloidal material (COD and silica) were obtained, which is required for extended reuse of this process water. PAC-HB was the best product for removing suspended solids (85%) and soluble silica (50%) with a rather limited COD removal (5%), while PANS-PA obtained high turbidity (90%) and silica removal (45%) together with a significant soluble COD removal (15%). Monomeric Al (Al_a_, Al_m_) was more efficient in removing suspended solids and soluble COD than polymeric or colloidal Al (Al_c_, Al_u_), but the latter was more efficient in removing soluble silica. Results demonstrated that the main flocculation mechanism varies with the aluminum dosage, being predominantly charge neutralization at low dosages and sweep flocculation at high dosages. The floc strength factor however, was very high and similar for all the coagulants and dosages tested (85–90%), as it was mainly determined by the behavior of the pre-flocculated suspended solids present in wastewater. The reflocculation factor varied from 45 to 75% at the lowest dosages to almost zero at the highest dosages, confirming the transition from charge neutralization to sweep flocculation. The flocs formed by PANS-PA had lower strength than the others and it decreased with the dosage while its reflocculation factor was almost zero, even at low dosages. Due to the polyamines present in this coagulant, its flocculation mechanism is through both charge neutralization and patch formation, especially at low dosages, and sweep flocculation and interparticle bridge formation at high dosages.

## Introduction

Dissolved air flotation (DAF) is a very efficient treatment technology for the removal of suspended matter, either oil and grease or solids (Edzwald and Haarhoff, 2011; Bolto and Xie, 2019). Internal water reuse after DAF is the most common alternative used in paper mills to reduce fresh water consumption (Hubbe et al., 2016). In recycled newsprint mills, there are up to three or four DAF units in each production line (one within each water loop). In these systems, suspended solids are efficiently removed but the dissolved and colloidal material (DCM) is almost completely recirculated into the process, accumulating in the water circuits, and thus limiting their closure (Miranda et al., 2009a; Hubbe et al., 2016; Ordaz-Díaz et al., 2017). However, by extending the limits of coagulation/flocculation by an improved understanding of the destabilization mechanisms it is possible to develop improved chemicals which are able to remove also finely dispersed and colloidal particles (>0.1–0.2 μm) by DAF. In these conditions, DAF units can remove 80–99% of suspended solids, together with, in the best cases, up to 10–30% of soluble chemical oxygen demand (COD) (Miranda et al., 2008, 2009b,c, 2013).

A variety of coagulants have been used in DAF systems including alum, ferric chloride, polyaluminum chloride (PAC), polyamine (PA), polydiallyldimethylammonium chloride (PDADMAC), chitosan, etc. (Ordaz-Díaz et al., 2017; Bolto and Xie, 2019). Aluminum based coagulants are generally the most versatile and widely used. In addition to alum, many types of polyaluminum coagulants are commercially available for water treatment such as aluminum chlorohydrate, PAC, and polyaluminum sulfates. These products differ in their basicity and strength, and can contain small amounts of other compounds such as sulfate, nitrate, silica, and calcium (Pernitsky and Edzwald, 2006; Edzwald and Haarhoff, 2011). Furthermore, polyaluminum coagulants can be used in composite coagulants in combination with PA, PDADMAC, polyacrylamides, bioflocculants, and even other inorganic coagulants, e.g., polymeric aluminum ferric silicates, to improve their performance (Lee et al., 2012; Chen et al., 2015; Huang et al., 2015; Tang et al., 2015; Latour et al., 2016; Sun et al., 2017; Wang X. et al., 2017). The distribution of aluminum species in polymeric aluminum coagulants influences their coagulation performance, however, the relationship is not well-established yet and depends on the exact wastewater and contaminants considered.

Understanding the flocculation mechanism is also of great importance as both the size and the structure of the formed aggregates affect the removal efficiency of contaminants (Lin et al., 2008). There are several possible flocculation mechanisms described in the literature including charge neutralization, sweep flocculation (flocculation by enmeshment), interparticle bridging and patch formation (Bache and Gregory, 2007; Bratby, 2016). The predominant flocculation mechanisms for alum and polyaluminum coagulants are charge neutralization and sweep flocculation (Edzwald and Haarhoff, 2011). Neutral conditions are considered favorable for aluminum-based coagulants due to the presence of positively charged Al species and to the fact that most of the Al is precipitated, forming floc particles [conditions of minimum solubility of Al(OH)_3_] (Pernitsky and Edzwald, 2006; Edzwald and Haarhoff, 2011). At these conditions, charge neutralization is the predominant mechanism at low aluminum dosages, while the precipitation of Al(OH)_3_ and the subsequent enmeshment of colloids in the precipitate predominates at high dosages. However, it is important to notice that the DCM destabilization cannot be exclusively attributed to a particular mechanism, especially when complex industrial waters are treated (Bratby, 2016). Apart from the nature and dosage of the coagulant used, there are also other factors determining the flocculation mechanisms such as the nature and the surface charge of the particles, the pH, the amount of colloidal and suspended solids, the ionic strength, etc.

There are many studies analyzing flocculation with aluminum salts, some of them analyzing floc strength under different shear forces (deflocculation studies) and the reversibility of the flocculation after breakage (reflocculation studies). However, these studies usually focus on model suspensions of kaolin (Cheng et al., 2011; Wang et al., 2011), humic acids (Wang et al., 2009), or a combination of both (Yu et al., 2009; Zhao et al., 2010; Yao et al., 2014; Nan et al., 2016; Wang Z. et al., 2017) and only in some cases with drinking waters (Yao et al., 2015; Jiao et al., 2016). However, the study of flocculation mechanisms in industrial wastewaters are very limited. Furthermore, there are only limited references regarding the flocculation mechanisms of hybrid or composite coagulants, most of them with iron-based hybrids (Wei et al., 2010; Zhang et al., 2015; Wang B. et al., 2017), and only a few recent studies with aluminum-based hybrid coagulants (Latour et al., 2016; Kangama et al., 2018; Shen et al., 2020).

The main objective of this paper is to analyse how to extend the removal of contaminants by DAF using different aluminum coagulants by understanding the influence of their speciation and flocculation mechanisms. The study is aimed specifically at treating process waters from the paper industry.

## Materials and Methods

### Materials

#### Water Samples

They were taken from the inlet of a DAF unit from a 100% recycled newsprint mill located in Madrid (Spain). The main characteristics of these waters are shown in Table 1.

**Table 1:** Characteristics of the DAF inlet waters and DAF blank.

	**DAF Inlet**	**DAF blank**
**Raw waters**		
pH	6.9 ± 0.2	6.8 ± 0.2
Conductivity (25°C) (mS/cm)	2.62 ± 0.18	2.12 ± 0.16
Total solids (mg/L)	5520 ± 185	3395 ± 165
COD (mg/L)	3665 ± 150	2332 ± 105
Total suspended solids (mg/L)	1620 ± 80	183 ± 20
Turbidity (NTU)	680 ± 45	268 ± 20
Cationic demand (meq/L)	1.16 ± 0.08	0.99 ± 0.07
Total alkalinity (mg/L CaCO_3_)	860 ± 60	645 ± 45
**After centrifugation**		
Total solids (mg/L)	3900 ± 160	3210 ± 140
Silica (mg/L SiO_2_)	273 ± 15	225 ± 12
COD (mg/L)	2600 ± 90	2050 ± 60
Turbidity (NTU)	21.8 ± 1.5	18.0 ± 1.4

*Average values and 95% confidence intervals were obtained from three replicates*.

#### Coagulants

Table 2 summarizes the properties of the tested aluminum-based coagulants. Alum [Al_2_(SO_4_)_3_·18H_2_O], reagent grade, was supplied by Panreac. PAC-MB is a conventional polyaluminum chloride with high aluminum content and intermediate basicity, and PAC-HB is a high-basicity polyaluminum chloride with intermediate aluminum content and a small amount of silica in its composition. On the other hand, PANS is a polyaluminum nitrate sulfate with an intermediate aluminum content and basicity, and PANS-PA is a hybrid coagulant obtained by the addition of a small amount of high charge quaternary polyamines of different molecular weights to PANS. PAC-MB, PANS, and PANS-PA were supplied by Sachtleben Wasserchemie GmbH while PAC-HB was supplied by Kemira Ibérica, S.A.

**Table 2:** Characteristics of the coagulants used in this study.

**Coagulant**	**Al_**2**_O_**3**_ (%)**	**Basicity (%)**	**Charge density (meq/g)**	**Density (g/cm^**3**^)**	**pH**	**Dry content (%)**
Alum	15.3	0	–	–	–	–
PAC-MB	16.8	37	1.77	1.37	<1	34.1
PAC-HB	9.7	85	1.67	1.22	2.7	29.5
PANS	10.2	46	1.22	1.27	2.6	21.7
PANS-PA	6.05	–	2.57	1.23	3.0	20.4

Furthermore, the aluminum species distribution of the used coagulants were measured by the ferron test and ^27^Al NMR spectroscopy (Table 3). Ferron test was carried out in duplicate according to the method described in Wang et al. (2004), with a total concentration of aluminum of 10^−5^ M and maintaining the ferron/Al_T_ molar ratio >10. In this method, Al_a_ denotes aluminum species that reacted with ferron instantaneously (within 1 min). Al_b_ denotes species that reacted within 120 min and finally, Al_c_ denotes species that did not react. These species are assumed to be monomeric, polymeric and colloidal aluminum species, respectively. A Varian Cary 50 UV-Visible spectrophotometer was used for the absorbance measurements at 366 nm in 1-cm quartz cells. ^27^Al NMR spectra of the coagulants were obtained in Bruker Avance 500 spectrometer at a frequency 130.318 MHz. A 3 mm internal diameter capillary tube was inserted into a 5 mm internal diameter sample tube. This capillary tube containing a 0.1 M solution of NaAlO_2_ in D_2_O was used as inner standard of deuterium lock and for Al quantification. In all the samples, the number of scans was fixed to 64, the pulse width to 7.0 μs and the temperature to 298 K. The resonance corresponding to the monomeric aluminum such as Al^3+^, Al(OH)^2+^, and Al(OH)^3+^, was assigned a chemical shift of 0 mg/L (Al_m_). The signal at 62.5 mg/L denotes ${{\text{Al}}_{13}{\text{O}}_{4}(\text{OH})}_{24}^{7+}$ (Al_13_ in short). Finally, the signal at 80.0 mg/L indicates the formation of ${\text{Al}(\text{OH})}_{4}^{-}$ from NaAlO_2_ used standard. Based on the integral areas of these three peaks, the aluminum concentration of the background and the sample, and the ratio of the cross-sectional areas of capillary to the sample tube, Al_m_ and Al_13_ were calculated. The undetectable aluminum (Al_u_) was obtained from the remaining Al_T_. Details for the calculations can be found in Gao et al. (2002). Previous studies have demonstrated that Al_a_, Al_b_, and Al_c_ species can be regarded approximately as Al_m_, Al_13_, and Al_u_, both methods obtaining similar Al species distribution (Hu et al., 2012).

**Table 3:** Distribution of coagulants aluminum species by ferron test and ^27^Al NMR for Al_T_ = 10^−5^ M.

	**Ferron test**			**^27^Al NMR**		
**Coagulant**	**Al_**a**_**	**Al_**b**_**	**Al_**c**_**	**Al_**m**_**	**Al_**13**_**	**Al_**u**_**
Alum	83.9	4.1	12.0	86.4	0	13.6
PAC-MB	54.5	44.3	1.3	36.9	50.0	13.1
PAC-HB	12.8	14.8	72.4	19.8	25.7	54.5
PANS	26.9	23.7	49.4	29.4	18.5	52.1
PANS-PA	23.2	24.4	52.4	25.1	20.7	54.2

The species distribution of PACl depends on preparing conditions such as basicity, total concentration of Al, rate of base addition, temperature, and so on. In general, Al_b_ and Al_13_ percentage in PACl increases with increasing basicity. The inclusion of sulfate in PACl did not largely influence aluminum species (Kimura et al., 2013). On the other hand, the presence of silica, for a constant basicity, usually imply lower shares of Al_m_ (Al_a_) and Al_13_ (Al_b_) but higher shares of Al_u_ (Al_c_) (Gao et al., 2002), which could justify why the Al_13_ (Al_b_) content of the highest basicity product is not the highest. Monomeric aluminum (Al_m_ or Al_a_) in alum (basicity = 0%) is usually 100%. In the present study, this content was slightly lower (around 85%) due to the formation of Al(H_2_O)_5_(SO_4_)^+^, evidenced by the presence of the resonance peak at −3.3 mg/L.

Coagulants in DAF tests were used in combination with an anionic polyacrylamide of high molecular weight and medium charge, supplied by SERTEC-20 S.L. (Spain). Dosages of the coagulants were selected according to preliminary tests with these waters, varying from 25 to 250 mg/L Al_2_O_3_, while the flocculant dosage was fixed to 10 mg/L in all cases.

### Methodology

#### DAF Tests

DAF tests were carried out in a laboratory-scale unit (Flottatest FTH3) supplied by Orchidis Laboratoires (France) using a sample volume of 1 L. For an easier comparison between the performance and flocculation mechanism of the coagulants, they were tested at the same equivalent Al_2_O_3_ dosage, from 25 to 250 mg/L Al_2_O_3_ (Chuang et al., 2007; Zhang et al., 2010), First, the coagulant was added from a 10% wt./vol. solution and mixed at high speed (180 rpm) for 2.5 min, and then the flocculant was added from a 0.10% wt./vol. solution and mixed at slow speed (40 rpm) for 10 min. Next, the flocculated waters were flotated adding a 20% tap water (200 mL) saturated with air at 7 bar. After 10 min flotation time, samples were collected from the bottom of the jars. A number of blanks were performed without adding any chemical before flotation, to consider both the dilution of the samples due to the addition of air-saturated tap water during flotation (20%) and also the physical efficiency of DAF to remove contaminants, even without chemical aids. The characteristics of these blanks, referred as 0 mg/L dosage, are included in Table 1. All experiments were carried out at room temperature (20–25°C) by duplicate, and the average error between replicates was ~5%. To avoid the possible water degradation, all tests and analyses were carried out within 5 days after sampling and the waters were always kept at 4°C before testing.

Clarified waters were characterized by pH, conductivity, turbidity, total solids, COD, cationic/anionic demand, and alkalinity. Furthermore, the dissolved fraction (obtained by centrifugation of clarified waters at 2,000 g for 15 min), was characterized by turbidity, total solids, silica, and sulfates. Total solids and turbidity were measured according to Standard Methods 2540B and 2130B, respectively, using a Hanna LP-2100 turbidimeter for turbidity. COD was measured by the Nanocolor® COD 1500 method from Macherey-Nagel GmbH, using an Aquamate Vis spectrophotometer (Thermo Scientific Inc.), according to ISO 15705:2003. Cationic/anionic demand was measured by colloidal titration using a Charge Analyzing System (CAS) supplied by AFG Analytic GmbH and polydiallyldimethylammonium chloride (PDADMAC) (0.001 N) and polyethylene sulfonic acid sodium salt (PES-Na) (0.001 N) as titrants. Dissolved silica was measured using the silico-molybddate method using flow injection analysis (FIA) according to ISO 16264:2002. Total alkalinity was measured by titration of the sample to pH = 4.5 with H_2_SO_4_ 0.1 N according to EPA 310.1 method. Sulfates were measured by the Nanocolor® Sulfate 200 or 1000 methods from Macherey-Nagel GmbH, using an Aquamate Vis spectrophotometer (Thermo Scientific Inc.). Finally, the pH and conductivity of the samples were analyzed using a GLP-22 pH-meter and a GLP-32 conductivity meter (both supplied by Crison Instruments, S.A.).

#### Flocculation Monitoring

Flocculation monitoring was carried out using a focused beam reflectance measurement (FBRM) system M500L, supplied by Mettler Toledo (USA), which measures in real time the chord length distribution of particles in suspension from 1 to 1,000 μm. From these data, total number of counts (TNC), mean chord size (MCS), counts in specific size intervals, and other statistical parameters can be easily calculated (Blanco et al., 2002a,b; Dunham et al., 2002), allowing the monitoring of flocculation, deflocculation, and reflocculation processes and thus, the characterization of the flocs properties and flocculation mechanisms. FBRM is able to study the flocculation process without the limitations of traditional methods such as charge titration, which are not valid when bridging or patching mechanisms dominate the system (Rasteiro et al., 2008). FBRM has been specifically used for the chemical optimization of DAF units treating thermomechanical pulp and deinked pulp waters (Saarimaa et al., 2006a,b; Miranda et al., 2008, 2013).

Two different flocculation studies were carried out: the flocculation tests by successive coagulant additions and the flocculation-deflocculation-reflocculation tests. In both cases, a sample of 250 mL was used. The flocculation by successive coagulant additions was carried out adding 25 mg/L Al_2_O_3_ of coagulant each 30 s using a 10% wt./vol. coagulant solution until a total dosage of 500 mg/L Al_2_O_3_ using 200 rpm stirring rate. In the flocculation-deflocculation-reflocculation tests, single dosages of coagulant, from 25 to 250 mg/L Al_2_O_3_, were used_._ First, the sample was stirred at 200 rpm during 30 s for stabilization and then the coagulant dosage was added from a 10% wt./vol. solution and the system was allowed to evolve for 2.5 min. After this time, the stirring rate was increased to 500 rpm and maintained during 2 min to break the formed flocs. Finally, the mixing speed was reduced to 200 rpm again and the system was monitored for other 5 min to analyse the reversibility of the flocculation. Mixing intensities used for flocculation and the breakage of the flocs stages were selected according to preliminary tests carried out with these waters.

The strength or breakage factor (SF) and recovery or reflocculation factor (RF) were calculated using Equations (1) and (2), where: MCS_1_ is the maximum MCS value before the flocs breakage, MCS_2_ is the MCS value when the flocs were broken after intensive stirring and MCS_3_ is the maximum MCS value for the flocs re-growth after the intensive stirring.

(1) $$\begin{array}{l} \text{SF}=\frac{{\text{MCS}}_{2}}{{\text{MCS}}_{1}} \end{array}$$

(2) $$\begin{array}{l} \text{RF}=\frac{{\text{MCS}}_{3}{-\text{MCS}}_{2}}{{\text{MCS}}_{1}{-\text{MCS}}_{2}} \end{array}$$

## Results and Discussion

### Removal of Contaminants by DAF

#### Turbidity

**Figure 1:**
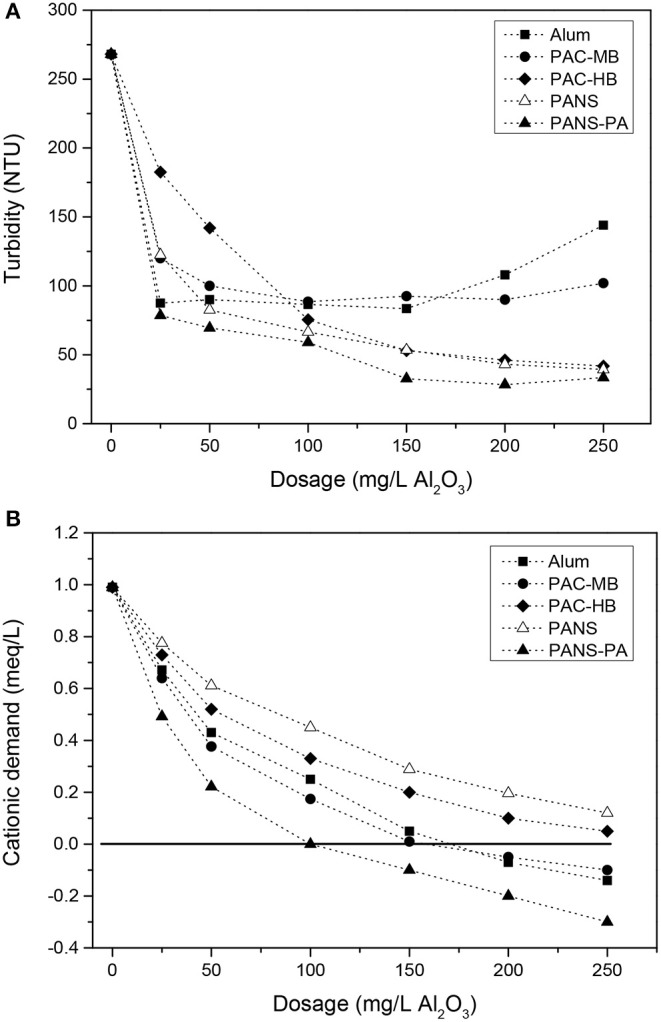
**(A)** Turbidity and **(B)** cationic demand of clarified waters vs. dosage of coagulants.

The initial size of the suspended solids is large enough to be partly removed by DAF. The mean chord size of the suspended solids was 41.7 μm while the distribution of particles among the different chord size intervals was: 34.6% (1–20 μm), 30.3% (20–50 μm), 26.3% (50–100 μm), and 8.9% (>100 μm) (FBRM measurements). Without chemicals, DAF reduced the turbidity from 680 NTU to 268 NTU (blank), which allows for the 20% decrease due to the dilution of raw waters with water saturated in air. The addition of a small amount of flocculant (10 mg/L) further reduced the turbidity to 196 NTU (27% removal referred to blank). However, coagulant addition was necessary to increase the efficiency. Turbidity removal increased greatly with coagulant addition but reached almost a constant value at dosages higher than 100–150 mg/L (Figure 1A). PANS, PANS-PA, and PAC-HB decreased the turbidity to a greater extent (30–40 NTU residual turbidity, 85–90% removal referred to blank) than alum and PAC-MB (80–90 NTU, 65–70% removal).

At the highest dosages of alum and PAC-MB (200–250 mg/L), charge reversal and restabilization was observed (see Figure 1B), producing an increase of the turbidity of the clarified waters. Although PANS-PA (the highest charged coagulant) reached charge reversal at lower dosages (100–150 mg/L), restabilization did not occur and turbidity removal did not decrease, which indicates a different flocculation mechanism for the hybrid coagulant. Charge reversal did not occur neither for PAC-HB nor PANS, the maximum reduction of cationic demand was 75% (final cationic demand of 0.25 meq/L).

The least efficient products were those having the highest monomeric Al content (Al_a_ or Al_m_) and the lowest basicities, thus producing the largest pH decreases after the treatment. Final pHs with these coagulants were lower than pH of minimum solubility of Al(OH)_3_, which is the most efficient pH to produce the largest amount of Al(OH)_3_ precipitates and the lower residual Al concentration in the clarified waters. Final pHs with alum and PAC-MB were around 0.5 pH units lower than the pH of minimum solubility, which is around 6.0 for alum and 6.2–6.4 for PACs, although the exact value depends on the wastewater composition (Pernitsky and Edzwald, 2003).

#### Soluble COD

**Figure 2:**
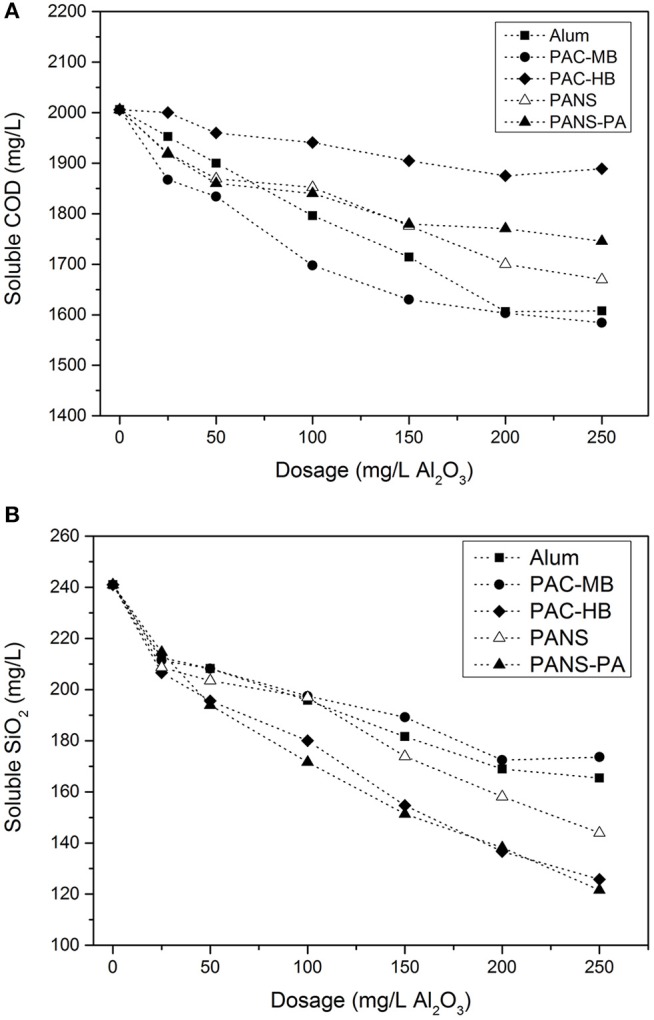
**(A)** Soluble COD and **(B)** soluble silica of clarified waters vs. dosage of coagulants.

For all the coagulation treatments, the higher the dosage the higher the soluble COD removal, however, at the highest dosages (200–250 mg/L Al_2_O_3_) only marginal COD removals were obtained (Figure 2A). The most efficient products were PAC-MB, alum, and PANS (20% removal). PANS-PA showed an intermediate efficiency (15% removal) and PAC-HB was the least efficient product (5% removal).

The soluble COD removal can be reasonably predicted by the species distribution of the coagulants. The best products in removing silica were those having the highest amount of monomeric Al (Al_a_ or Al_m_) except PANS, and the least efficient product (PAC-MB) was the one with the lowest monomeric Al. This means sweep flocculation, enhanced by high contents of monomeric Al, is more efficient than high contents of Al_b_ or Al_13_ for COD removal, which usually are more efficient for removing contaminants by charge neutralization and interparticles bridging mechanisms (Li et al., 2006).

#### Soluble Silica

Other parameter of interest in these process waters is soluble silica concentration. High levels of silica produce deposits in the process, impairing both the operational performance and the quality of the final product. Most important, the technical and economic feasibility of effluent reuse schemes by reverse osmosis treatments is limited by severe silica scale on membranes (Ordóñez et al., 2010). Thus, it would be very interesting to use the existing DAF units to partly remove the silica content in process water and thus in the effluent, especially taking into account that it has been previously demonstrated that silica removal by coagulation can be enhanced by the presence of suspended solids (Miranda et al., 2015a).

Silica removal increased with the coagulant dosage (Figure 2B). PAC-HB and PANS-PA were the most efficient products, with a 45–50% silica removal (125–130 mg/L residual silica), while PANS, PAC-MB, and alum, obtained a 30–35% removal (155–175 mg/L residual silica). The soluble silica removal can be related to the content of colloidal aluminum (Al_c_ or Al_u_) in the coagulants. Most efficient products (PAC-HB and PANS-PA) were those with the highest contents while the least efficiency products (alum and PAC-MB) are those with the lowest ones. This is in agreement with a previous work studying the effect of aluminum speciation on silica removal during the coagulation of heavy-oil wastewater using polyaluminum chlorides (Zhao et al., 2016). These authors demonstrated that Al_a_ and Al_c_ promoted the removal of Si_c_ (polymeric) and Si_a_ (monomer and dimers), while they did not find a clear trend for Al_b_. In the present study, most silica species are monomers and dimers of silicilic acid (Si_a_) as demonstrated by the fact that the silica concentration measured by silico-molybdate method was around the same than the total silica measured by ICP-OES: 273 mg/L vs. 277 mg/L SiO_2_. Thus, present study corroborates Al_c_ is the most important Al species for the removal of soluble silica consisting in monomer and dimers.

Previous studies have demonstrated that the coagulants with the highest efficiency in removing COD were usually those with the lowest efficiency in silica removal, and vice versa (Hermosilla et al., 2012; Latour et al., 2013; Miranda et al., 2015b). However, the reason for this was not established. According to the results obtained in this study, the explanation seems to be related to Al speciation: coagulants with high Al_a_ (Al_m_) content are more efficient in removing soluble COD while coagulants with high Al_c_ (Al_u_) content are more efficient in removing soluble silica. This is very important as the coagulant type and dosage used in industrial DAFs is usually optimized in terms of suspended solids (turbidity). However, the removal of soluble contaminants is required to further closing the water circuits.

#### pH and Conductivity

These parameters are also important when reusing process waters. When added to water, aluminum ions hydrolyse to form soluble monomeric and polymeric species and solid precipitates, which causes an alkalinity consumption and a parallel pH decrease, the extent of this decrease depends on the aluminum dosage and the basicity of the coagulants (Pernitsky and Edzwald, 2006). As the same aluminum dosages have been tested for all the coagulants, the highest pH decrease was observed for the products with the lowest basicity. Alum and PAC-MB decreased the pH of clarified waters to pH 5.4 and 6.0, respectively, at the highest dosage tested. On the opposite, PAC-HB and PANS-PA decreased less the pH of the treated waters, this decrease being always lower than 0.5 pH units (final pH 6.5–6.8 at the highest dosage). The pH decrease with PANS was intermediate. As commented before, the pH of minimum solubility of Al(OH)_3_, which produces the larger amount of Al(OH)_3_ and the lower residual aluminum concentration, is around 6.0 for alum and 6.2–6.4 for PACs (Pernitsky and Edzwald, 2003). For this reason, the lowest pH at which aluminum salts can be used is usually limited to around 5.5–5.8, depending on the temperature and the presence of other species in the waters, i.e., sulfates, phosphates, etc. (Kvech and Edwards, 2002). Accordingly, for the treatments based on alum or PAC-MB, especially if high dosages are used, it would be recommended to add alkalinity to the waters.

Similarly to pH, PAC-HB, and PANS-PA were the products that induced a lower increase of conductivity, from 2.1 mS/cm (blank) to 2.4–2.5 mS/cm. The other coagulants increased the conductivity of the treated waters to 2.8 mS/cm (PANS and PAC-MB) or 2.9 mS/cm (alum); all these values were obtained at the highest dosages tested.

It is also important to notice that the distribution of aluminum species depends primarily of the coagulation pH. When added to water, aluminum ions hydrolyse to form soluble monomeric and polymeric species and solid precipitates, the main ones being Al^3+^, Al(OH)^2+^, ${\text{Al}(\text{OH})}_{2}^{+}$, Al(OH)_3_ (s), ${\text{Al}(\text{OH})}_{4}^{-}$, ${{\text{Al}}_{2}\text{OH})}_{2}^{4+}$, ${{\text{Al}}_{3}(\text{OH})}_{4}^{5+}$, ${{\text{Al}}_{13}{\text{O}}_{4}(\text{OH})}_{24}^{7+}$, and Al(OH)_3_ (s). Under normal water treatment conditions, alum chemistry can be described by the presence of three species: ${\text{Al}({\text{H}}_{2}\text{O})}_{6}^{3+}$ (usually shortened to Al^3+^), Al(OH)^2+^, and Al(OH)^4−^ in equilibrium with an amorphous Al(OH)_3_ solid phase. Below the pH of minimum solubility, the highly charged Al^3+^ and Al(OH)^2+^ species are the most prevalent species, while at greater pHs, become predominant ${\text{Al}(\text{OH})}_{4}^{-}$ (Pernitsky and Edzwald, 2006; Edzwald and Haarhoff, 2011). The solid phase formed upon precipitation, Al(OH)_3_ (s), has amphoteric hydroxyl groups that can charge positively or negatively depending on the pH. The chemistry of PACs is very similar to that of alum but PACs contain highly charged polymeric aluminum species in addition of the monomers described for alum, the most predominant being ${\text{Al}}_{13}^{7+}$, which is more stable in a wide range of pHs than other polymerized ${\text{Al}}_{13}^{7+}$ is more stable in a wider range of pH. The basicity of PACs affects the alkalinity consumption of the coagulant but also the relative prevalence of polymeric and monomeric species. In general, the higher basicity, the greater ${\text{Al}}_{13}^{7+}$ fraction, up to ~70% basicity (Pernitsky and Edzwald, 2006). As a rule of thumb, the best efficiency is obtained by coagulation near the pH of minimum solubility (pH from 6 to 7) where the amount of aluminum hydroxide precipitated is the largest, while keeping the Al residual levels in water at minimum. If coagulation pH is low or pH suppression has been important, e.g., coagulation at pHs 5.5 or less, then the positively charged Al species become predominant. This fact together with the higher Al dissolved levels make the primary mechanism for the removal of contaminants complexation and direct precipitation (Edzwald and Haarhoff, 2011).

#### Comparison Among the Treatments

Comparing the obtained results for turbidity, COD, and silica removal, PANS-PA and PAC-HB, those which also produced the lowest pH decrease and conductivity increase, were the most efficient treatments. PAC-HB will be the recommended treatment if the most important objective of the treatment is suspended solids and soluble silica removal, while PANS-PA would be better option for obtaining high suspended and soluble silica removal together with an intermediate COD removal. An interesting fact to notice is that different aluminum compounds can be selected depending on the exact needs of the plant, i.e., tailor-made solutions.

Dissolved air flotation is a very efficient technique for the removal of suspended solids. However, the combination of DAF with a suitable coagulation chemistry has demonstrated it is also possible to important removals of dissolved contaminants such as COD and silica, even when most of the initial suspended solids were lower than 50 μm.

The comparison of the results obtained with data from the literature is challenging as the removal efficiencies in these treatments depend largely of the exact water treated as well as the coagulation chemicals and conditions tested (pH, dosages, etc.). It has been previously demonstrated that even in the same paper mill, using the same chemicals, important efficiency differences are found depending on the process water quality (Miranda et al., 2015a). Table 4 shows the most relevant studies found in the literature using similar chemicals and treating similar waters. It is observed that the chemicals tested in the present study were similar in the removal of turbidity and soluble COD, to other products however, they also allowed a high removal of soluble silica, which was one of the main objectives of this study. This is an important achievement because an inverse relationship between soluble COD and silica removal is usually found (Latour et al., 2013).

**Table 4:** Comparison of the results obtained for the removal of contaminants by coagulation with relevant literature.

**Water type**	**Removal of contaminants obtained**	**References**
Process waters from recycled paper production	PANS-PA: 90% turbidity, 45% soluble silica, and 15% soluble COD. PAC-HB: 85% turbidity, 50% soluble silica, and 5% soluble COD. Alum, PAC-MB, and PANS: 65–80% turbidity, 30–35% soluble silica, and 20% soluble COD.	Present study
Process waters from recycled paper production	DAF1 waters: PANS-PA, PAC-HB, and PANS removed 80–85% turbidity, 25–35% soluble silica, and 5–20% soluble COD. Alum and PAC-MB removed 65–70% turbidity, 20% soluble silica, and 20% soluble COD.	Miranda et al., 2015a
	DAF2 waters: PANS-PA, PAC-MB, and alum removed 95% turbidity, 20–35% soluble silica, and 20–25% soluble COD. PANS and PAC-HB removed 65–80% turbidity, 20–25% soluble silica, and 10–20% soluble COD.	
Effluent from recycled paper production	Newly developed hybrid coagulants combining PANS and three polyamines of different molecular weights and similar charge densities. Without pH adjustment (pH 8.4), the following removals were obtained: >75% turbidity, 40–50% soluble silica, and up to 40% soluble COD.	Latour et al., 2016
Process waters from recycled paper production	Different coagulation treatments, including bentonite + anionic polyacrylamide, PDADMAC, or PAC. The following contaminants removal were obtained: 60–90% turbidity, 5–50% COD, 0% silica.	Sarja et al., 2004
Process waters from recycled paper production	Different polyaluminum based coagulants (including hybrid coagulants) obtained 90% removal of turbidity and 15% of soluble COD.	Miranda et al., 2009a
Process waters from recycled paper production	Dual treatment (chitosans + anionic bentonite microparticles): 80–90% turbidity, 5–10% soluble silica, and 20% soluble COD.	Miranda et al., 2013
The secondary treatment effluent from a high-tech industrial park	PAC/alum coagulation in combination with waste flocs and polyacrylic acid flocculant achieved the following removals at optimal conditions: 95% removal of turbidity, 45–50% colloidal and soluble silica, and 60% soluble COD.	Chuang et al., 2007

### Flocculation Studies

#### Flocculation by Successive Coagulant Additions

**Figure 3:**
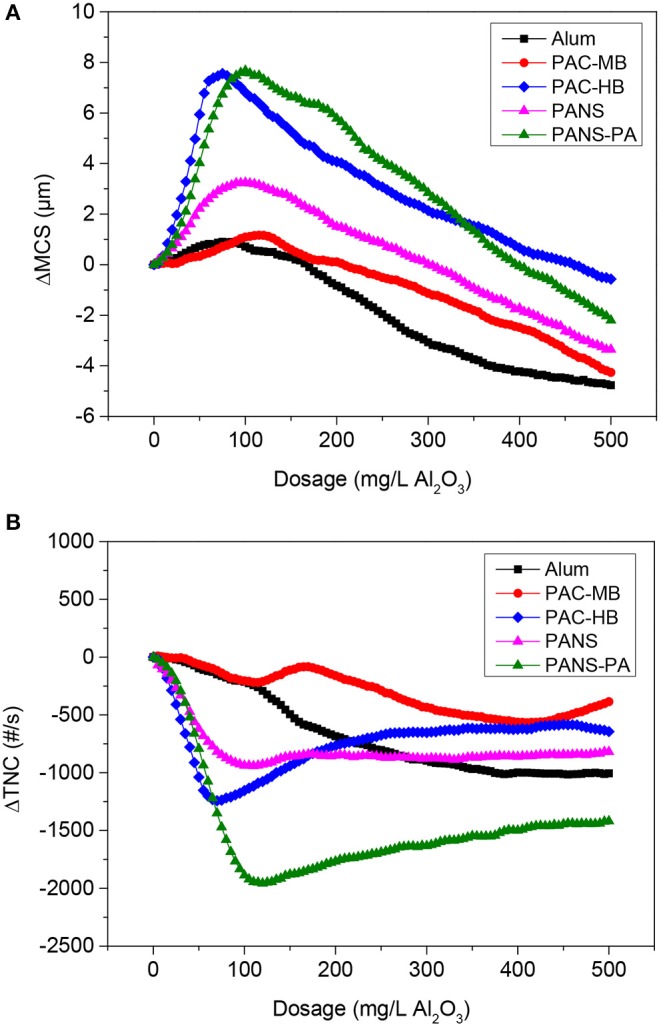
**(A)** ΔMCS and (B) ΔTNC vs. dosage of coagulants without flocculant addition (MCS_o_ = 41.7 μm, TNC_o_ = 2,860 #/s).

As the coagulant dosage increased, the total number of counts (TNC) decreased and the mean chord size (MCS) increased, indicating an increased aggregation of the particles. This behavior was observed until reached an optimal dosage and no more aggregation took place after: TNC stopped decreasing and started to increase, while MCS stopped increasing and started to decrease due to steric stabilization or electrostatic repulsion (Figure 3).

Two products stood out from the rest, PANS-PA and PAC-HB. At their optimal dosages, they reduced notably the initial TNC (70% reduction for PANS-PA and 45% for PAC-HB) while increased around 8 μm the MCS of the particles. PANS shown an intermediate efficiency both in terms of MCS increase (3 μm) and TNC reduction (35%). Finally, PAC-MB and alum were the coagulants increasing less the MCS (around 1 μm); PAC-MB was the coagulant producing also the lowest TNC decrease (20%), while alum obtained an intermediate TNC reduction (35%).

The important decrease of the initial TNC is in agreement with the high efficiency in the removal of contaminants in DAF tests. For example, turbidity removals varied from 65 to 70% with the least efficient products to 85–90% with the most efficient products. Despite an extensive flocculation was observed during these flocculation tests, the observed MCS increases obtained by FBRM could be considered small. However, this is explained by the following two facts. First, floc larger than 1,000 μm, visually observed during the flocculation, are not detected by the FBRM probe (measurement range from 1 to 1,000 μm). Second, the important destabilization of DCM (as demonstrated by the high removals of soluble COD and silica) forms new small flocs, previously not detected by FBRM, which decreases the MCS of the present particles.

The dosage at which the maximum MCS or the minimum TNC was observed can be used as rough estimation of the optimal coagulant dosage. In this case, the optimal dosages ranged between 75 and 125 mg/L Al_2_O_3_ for all the coagulants tested. These preliminary optimal dosages are in agreement with those observed for turbidity removal in DAF tests (Figure 1A), as the flocculation of suspended solids is the main contributor of turbidity. However, as commented before, the maximum removal of soluble COD or silica was only achieved at the highest dosages tested. As soluble COD and silica are not associated with suspended solids, a poor relation between ΔMCS and ΔTNC and COD and silica removal was observed.

#### Flocculation-Deflocculation-Reflocculation Studies

**Figure 4:**
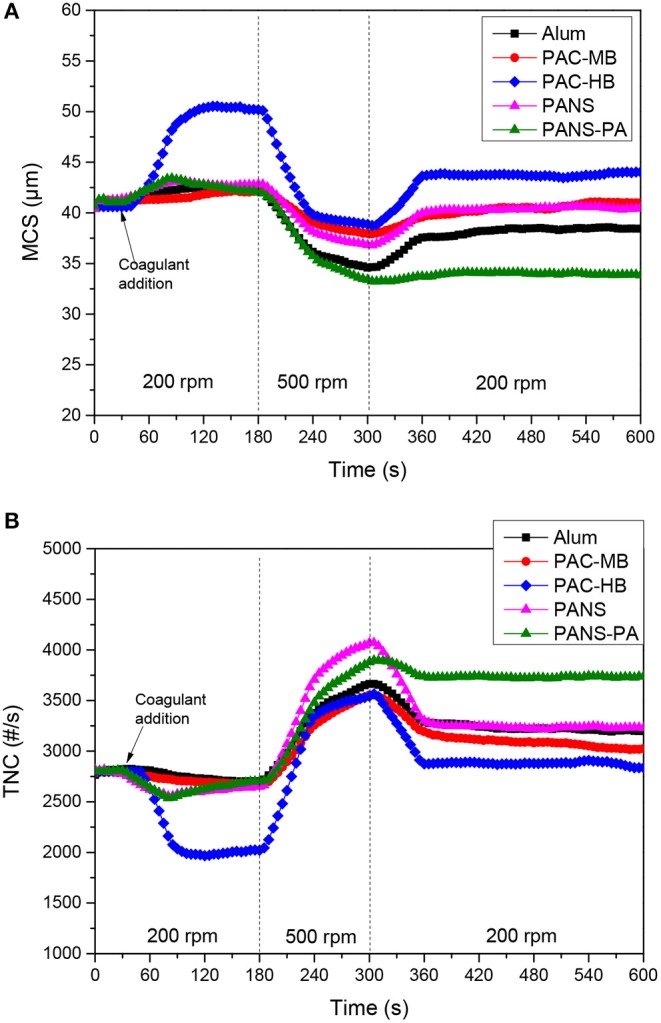
**(A)** MCS and **(B)** TNC at 25 mg/L Al_2_O_3_ coagulant vs. time in flocculation-deflocculation-reflocculation studies.

**Figure 5:**
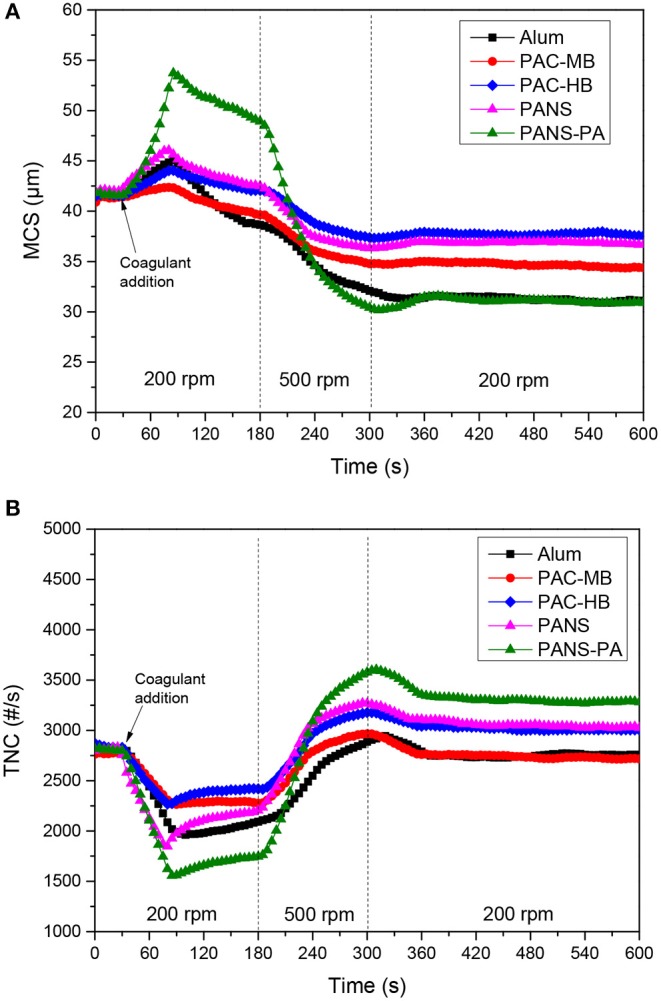
**(A)** MCS and **(B)** TNC at 250 mg/L Al_2_O_3_ coagulant vs. time in flocculation-deflocculation-reflocculation studies.

Growth, breakage and regrowth of the flocs formed by coagulation with different dosages of the aluminum compounds were studied. As the observed behavior showed a continuous transition from the lowest (25 mg/L) to the highest (250 mg/L) dosages tested, the flocculation-deflocculation-reflocculation curves only for these two dosages are shown in Figures 4, 5.

##### Flocculation stage

At low dosages (25–50 mg/L) neither the MCS increased nor the TNC decreased significantly except for PAC-HB. However, at higher dosages (≥100 mg/L), a significant increase in the MCS and a significant decrease in TNC were observed for all the coagulants, indicating an important agglomeration of the particles. The extent of this agglomeration increased with the dosage for all the coagulants except for PAC-HB. At the lowest dosage tested (25 mg/L), PAC-HB was the coagulant increasing in a larger extent the MCS (around 10 μm compared to 1.0–1.5 μm for the other coagulants) and decreasing the TNC (850 #/s compared to 100–250 #/s for the other coagulants). However, at the highest dosage tested (250 mg/L), PANS-PA produced the highest increase in the MCS (around 12 μm compared to <4 μm for the other coagulants) and the highest decrease in TNC (1,400 #/s compared to 500–1,000 #/s).

According to these results, PANS-PA seems to be the most efficient product, especially at intermediate and high dosages, while PAC-HB would be the most efficient at low dosages. However, PAC-HB was one of the most efficient coagulants in DAF tests (especially in turbidity and soluble silica removal) and its efficiency always increased with the dosage, with no apparent restabilization or charge reversal according to turbidity and cationic demand measurements. The reason for this apparent controversy is related to the shape of the flocs formed by PAC-HB. A previous study of the authors demonstrated this coagulant can induce the linear aggregation of the particles, especially at high dosages, generating cylindrical coagula (Hermosilla et al., 2012). These coagula are much longer than the original particles but with similar diameter. As the probability of the laser beam of FBRM to intercept the particle at the longer side is much lower than at the shorter side, this aggregation was not directly observed by FBRM.

The other three coagulants gave similar results from flocculation monitoring tests, the efficiency of PANS being slightly higher than that of alum and PAC-MB, which is also in agreement with the observed removal of contaminants in DAF tests.

##### Deflocculation stage and strength factor

**Figure 6:**
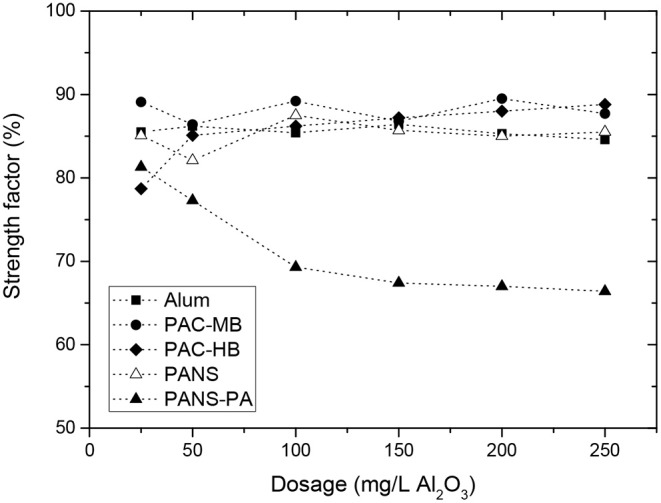
Strength factor for the different coagulants and used dosages.

The observed behavior was very similar for all the coagulants but PANS-PA. During the deflocculation of flocs formed by pure aluminum coagulants, the MCS decreased 4–8 μm while the TNC increased 750–1,500 #/s. The MCS decrease and TNC increase of the flocs formed by PANS-PA was higher and varying largely with the dosage: the MCS decrease varied from 8 μm at 25 mg/L to 18 μm at 250 mg/L, while the TNC increase varied from 1,800 #/s at 25 mg/L to 2,500 #/s at 250 mg/L. The differences in the strength of the formed flocs was further analyzed by the strength factor (Figure 6). The strength factor for pure aluminum coagulants was high (most of them between 70 and 75%), indicating the flocs formed were very resistant to shear forces, and only small differences with the coagulant type and dosage was observed. The strength factor of PANS-PA was, however, lower than those obtained by pure aluminum coagulants and decreased significantly with the dosage, from around 80 to 65%.

However, flocs formed by charge neutralization or sweep flocculation, which are the two main flocculation mechanisms associated to aluminum based coagulants, are weak and prone to breakage (Bache and Gregory, 2007; Ghernaout and Ghernaout, 2012; Bratby, 2016). Although the strength factor is dependent on the breakage shear rate and the mixer characteristics (Jarvis et al., 2005), a review of the literature indicates that regular strength factors for charge neutralization and sweep flocculation mechanisms are much lower than those obtained in the present study. Xu and Gao (2012), for example, obtained strength factors around 50–65% for different aluminum coagulants at low shear intensities (breakage at 100 rpm during 5 min) and values from 10 to 25% at high shear intensities (breakage at 400 rpm during 5 min).

The high strength factors obtained in this study are similar to those obtained by other flocculation mechanisms such as interparticles bridges formation. This apparent controversy can be explained taking into count the effect of shear forces on the initially present suspended solids in wastewater. The wastewater tested is the filtrate from a gravity table used to thicken the sludge rejects from different process stages. To improve the sludge dewatering, the sludge was flocculated before the gravity table, thus the suspended solids initially present in the wastewater are partially flocculated.

**Figure 7:**
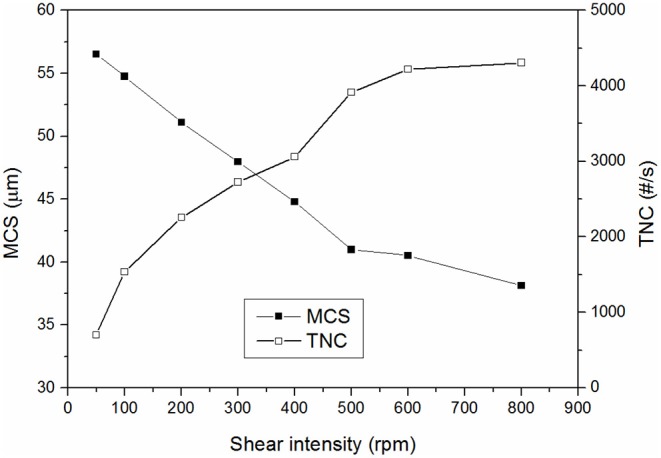
Effect of shear intensity on MCS and TNC of the initial water.

Figure 7 shows the effect of shear forces on the MCS and TNC of the wastewater without adding any coagulant. As it can be observed, the shear forces had a large impact on the TNC and MCS of the initially present suspended solids. Additionally, deflocculation-reflocculation tests were also carried out at the same experimental conditions and the MCS of the initial solids decreased 14.3 ± 1.7 μm and the TNC increased 230 ± 20 #/s, giving a strength factor of around 80 ± 2% (three replicates). This high strength factor is in agreement with interparticles bridges formation mechanism, which typically occurs when high molecular weight and low charge polyelectrolytes are used, which is exactly the characteristics of the cationic polyacrylamide used as flocculant in the gravity table. This justifies the high strength factors obtained in the present study as the observed flocs strength was mainly determined by the strength of the pre-flocculated suspended solids which were initially present in the wastewater. The flocs formed by aluminum coagulants only reduced the strength of the flocs from around 80% to 70–75%, in agreement with the expected lower strength of the flocs formed by either charge neutralization or sweep flocculation mechanisms.

From the obtained results it is difficult to distinguish if the main flocculation mechanism for aluminum coagulants was charge neutralization or sweep flocculation. First, the strength factors were very similar for all the treatments tested and second, it is not clear from the literature which flocs are more resistant to shear forces. In these sense, some authors have obtained a higher strength for the flocs formed by charge neutralization than those formed by sweep flocculation (Li et al., 2006; Wang et al., 2009), while others have obtained the opposite result (Gregory and Duan, 2001). This is probably because the flocs produced by precipitation are of widely varying strength and density (Bratby, 2016). In general, charge neutralization typically occurs at low pHs (pH<6.5) and low ionic strength waters. Sweep coagulation, in contrast, occurs principally at near neutral pH and at coagulant dosages >0.05 mmol Al/L (2.5 mg/L Al_2_O_3_) (Jiang and Graham, 1998). The experimental conditions at which the coagulants were tested are the optimal for sweep flocculation (pH near neutral and high conductivity and soluble organics), especially at high coagulant dosages. However, charge neutralization may be also taking place at the lowest dosages tested, especially for the pre-polymerized coagulants, as it will be demonstrated clearer with the analysis of the reversibility of the flocs breakage.

It is also difficult to distinguish different behavior from different aluminum coagulants. In general, PACl products give slightly stronger flocs than alum at equivalent aluminum dosages (Jarvis et al., 2005). However, in the present study, the strength of alum flocs was similar than for PACls. PAC-HB was the only coagulant showing a slightly different behavior than the rest of PACls. In this case, the strength factor was around 10–15 points lower at 25 mg/L than those of the other coagulants. This coagulant was the one with the lowest Al_a_ (Al_m_) content and thus producing the lowest amount of Al(OH)_3_. Previous studies have demonstrated that monomeric aluminum produced strongest flocs than the flocs formed by PACl with lower amount of Al_m_ and higher contents of Al_13_ and Al_c_ in the flocculation of humic acids (Wang et al., 2009), thus this could be the explanation. Furthermore, PAC-HB was the coagulant achieving the highest increase in the MCS at 25 mg/L (10 μm increase vs. 1.5–2.5 μm for other coagulants) and the floc strength increases with decreasing flocs size (McCurdy et al., 2004). For these two facts, it is reasonable PAC-HB had a slightly lower floc strength than the other coagulants, especially at low dosages.

Flocs formed by PANS-PA were weaker than those formed by pure aluminum compounds, which could be explained by the different flocculation mechanism but also taking into account that the formed flocs by PANS-PA were the largest before the deflocculation stage, especially at high dosages, and larger flocs are usually the weakest (Bache and Gregory, 2007). It was observed a negative correlation between the strength factor and size of the flocs after coagulation. The highest strength factors were observed at the lowest dosages, when the lowest MCS increases after coagulation were observed (7–9 μm), and then decreased and maintained constant at the dosages at which the highest MCS were observed (17–18 μm). PANS-PA flocculation mechanism is a combination of the main flocculation mechanism of PANS and that of the polyamines used in its formulation. Depending on its molecular weight and charge density, the flocculation mechanism of polyamines is usually explained by patches formation or interparticles bridging formation. The flocs formed by patches are generally soft, small and rigid, while the flocs formed by bridges are big, hard, and flexible. If the main flocculation mechanism would be bridges formation, the strength factor should be higher than those for pure aluminum coagulants, while if patches formation is predominant, the strength factors should be very similar to that for pure aluminum coagulants. PANS-PA had strength factors similar to that of pure aluminum coagulants at 25 mg/L but much lower at higher dosages. High charge and low molecular weight polyamines as those used in the PANS-PA formulation usually works by patches formation, which could explain the similar strength factor of PANS-PA to those of the other coagulants at 25 mg/L. The weaker flocs obtained at high dosages can be explained by the impaired formation of patches at these conditions, which is in agreement with the high cationic demand removals obtained with this product and the charge reversal obtained at dosages ≥100 mg/L. Furthermore, the flocs obtained were larger and were associated to higher removal efficiencies of contaminants at high dosages. Therefore, flocculation mechanism is a combination of sweep flocculation by PANS and the formation of large and loose organic flocs induced by polyamines.

##### Reflocculation stage and reflocculation factor

**Figure 8:**
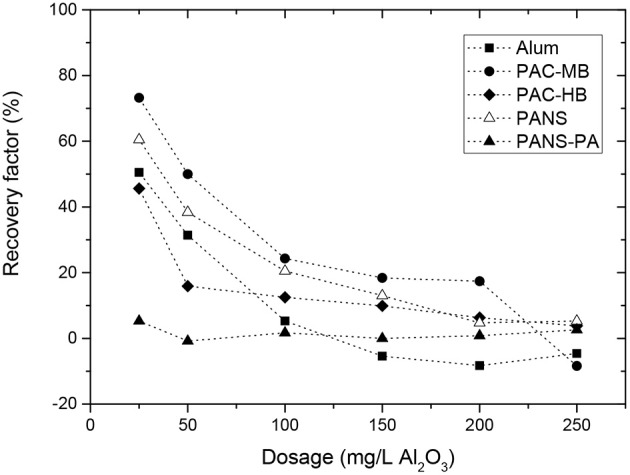
Reflocculation factors for the different coagulants and used dosages.

For all the coagulants and dosages tested, the reflocculation was very limited, especially for PANS-PA. In general, the higher dosage the lower MCS increase after the shear forces stopped and thus, the reversibility of the flocs. For pure aluminum coagulants, the MCS increase after reflocculation was 3–5 μm at low dosages (25–50 mg/L) and then decreased to 0–1 μm at the highest dosages. With PANS-PA, the reflocculation was almost negligible, even at the lowest dosage tested. Similarly to strength factors, reflocculation factors were calculated (Figure 8). Recovery factors decreased largely with the dosage for the pure aluminum-based coagulants, the main differences found at the lowest dosage tested. At 25 mg/L, the recovery factors varied in the following order: PAC-MB (73.2%) > PANS (60.5%) > alum (50.5%) > PAC-HB (45.6%) > PANS-PA (6%), while at the highest dosage (250 mg/L), recovery factors only varied between −8% (PAC-MB) and 5% (PANS).

At this point it is also interesting to take into account the reflocculation factor obtained without adding any coagulant, which shows the behavior of the initially present suspended solids: this value is 25.7 ± 1.5% (three replicates). This agrees well with the fact that initially suspended solids are partially flocculated by interparticles bridges (flocs of high strength but very limited recovery). Consequently, the recovery factors of the flocs formed by all coagulants but PANS-PA were greater than those from the initially present suspended solids. If charge neutralization would be the predominant flocculation mechanism, the reflocculation should be almost total after the shear forces ends (Blanco et al., 2002a,b). However, if sweep flocculation is predominant, the flocs will likely have a reduced recovery compared to charge neutralization (Gregory and Duan, 2001). The continuous decrease of the reflocculation factors with the dosage, from 45–75 to 0%, can be explained by the continuous transition from charge neutralization to sweep flocculation mechanism. At the lowest dosages, the contribution of charge neutralization mechanism is still important and high recovery factors are observed. As the dosage of the coagulant increases, sweep flocculation (irreversible flocs) became predominant, and a parallel decrease in the reflocculation factor of the formed flocs was observed. At the highest dosages (100–250 mg/L), the reflocculation factor was even lower than this obtained for the initially present suspended solids, thus indicating sweep flocculation became predominant.

The reflocculation factor was always close to zero for PANS-PA, independently of the dosage. The polyamines in its formulation could induce a partial patching mechanism, which would generate flocs that are partially reversible. These flocs are less reversible than charge neutralization but still more reversible than sweep flocculation. However, at the highest dosages, when charge reversal occurred, it is expected a transition from patches formation (reversible flocs) to interparticles bridges formation (irreversible flocs), which agrees well both with the larger flocs obtained by PANS-PA during flocculation and the null reversibility of the flocs, typical situation for interparticles bridge formation flocculation mechanism. Furthermore, the higher concentration of suspended solids after deflocculation favors interparticles bridging formation instead of patches (Tripathy and De, 2006; Zhou and Franks, 2006; Lee et al., 2014). This behavior would be explained by an increase in the contribution of interparticles bridges formation at the reflocculation conditions, where the high concentration of small solids promote interparticles bridges instead of patches formation.

## Conclusions

Aluminum coagulants were very efficient in removing contaminants by DAF, not only suspended solids but also soluble COD and silica. Large differences were found depending on coagulant composition and flocculation mechanism, which depends on the used dosage. Alum and PAC-MB were the least efficient coagulants, PANS had an intermediate efficiency, and PAC-HB and PANS-PA were the most efficient. The optimum coagulation treatment depends on the target contaminant, which depends on the particular needs of the industry. PAC-HB will be the recommended treatment when the most important objective of the treatment is suspended solids and silica removal, while PANS-PA is the recommended treatment for obtaining high silica removals together with high turbidity and COD removals. High Al_a_ (Al_m_) content of the coagulants is translated into high soluble COD removals but low or intermediate silica removals. On the other side, high Al_c_ content in the coagulants produced high silica removal but low or intermediate soluble COD removals.

Regarding flocculation mechanisms by pure aluminum coagulants, there is a continuous transition between charge neutralization and sweep flocculation. At the lowest dosages tested (25–50 mg/L), charge neutralization is the main flocculation mechanism while sweep flocculation is the main flocculation mechanism at the higher dosages (≥100–150 mg/L). The hybrid coagulant showed a rather different behavior result of the combination of the aluminum and the polyamines present, these polyamines acting by patches formation at low dosages (with a reduced shear resistance) and interparticles bridges formation at high dosages (confirmed by the large flocs observed and the limited reflocculation ability after flocs breakage). Strength and recovery factors were clearly affected by the behavior of the initially present suspended solids, which were flocculated in a previous stage (sludge thickening) by a high molecular weight and low charge flocculant in a process stage before sampling. These pre-existing flocs formed by interparticles bridging mechanism, characterized by high strength and null reversibility, modified the resulting flocs by aluminum coagulants which shown high strength and low reversibility after breakage, even at the lowest dosage when charge neutralization mechanism is predominant. As it was observed, the flocculation mechanisms taking place treating industrial waters is rather complex due to the large number of variables affecting the coagulation, including the presence of pre-flocculated suspended solids. However, these studies are necessary to get a deeper understanding of the flocculating mechanisms in such real systems.

The obtained results have demonstrated the different role of Al species of polyaluminum chlorides for the removal of suspended solids and soluble contaminants (COD and silica). This is an interesting starting point to develop tailor-made coagulants for target contaminants in other industrial applications. In the papermaking sector, it has been specifically demonstrated that the selection of an adequate chemistry allows existing DAF units to be optimized for removing specific contaminants even simultaneously if hybrid coagulants are used.

## Data Availability Statement

The datasets generated for this study are available on request to the corresponding author.

## Author Contributions

RM and IL made the experimental tests and the analysis of the results. RM wrote the first draft of the manuscript. All authors contributed to the conception and design of the study, manuscript revision, read and approved the submitted version.

### Conflict of Interest

The authors declare that the research was conducted in the absence of any commercial or financial relationships that could be construed as a potential conflict of interest.
